# Genotypes and Hot Spot Mutations of Hepatitis B Virus in Northwest Chinese Population and Its Correlation with Diseases Progression

**DOI:** 10.1155/2019/3890962

**Published:** 2019-12-10

**Authors:** Wei Wang, Yi Shu, Han Bao, Wenliang Zhao, Weihua Wang, Qin Wang, Xiaoying Lei, Daxiang Cui, Zhen Yan

**Affiliations:** ^1^The State Key Laboratory of Cancer Biology, Air Force Military Medical University, Xi'an 710032, China; ^2^Department of Biopharmaceutics, School of Pharmacy, Air Force Medical University, Xi'an 710032, China; ^3^Department of Pharmaceutics and Pharmacy Administration, School of Pharmacy, Air Force Medical University, Xi'an 710032, China; ^4^Department of Bio-Nano Science and Engineering, Key Laboratory for Thin Film and Microfabrication of Ministry of Education, Institute of Micro-Nano Science and Technology, Shanghai Jiao Tong University, Shanghai 200240, China

## Abstract

Hepatitis B virus (HBV) infection is a critical incentive for chronic hepatitis B (CHB), liver cirrhosis (LC), and hepatocellular carcinoma (HCC). Different genotypes and genome mutations of HBV have been found to be related to the progression of these liver diseases. However, their clinical significance is still under debate. The objective of this study was to determine the association of HBV genotypes and hot spot mutations in the reverse transcriptase (RT) and basal core promoter-precore (BCP-PreC) region with HBV-infected diseases in a northwest Chinese population. HBV genotyping and DNA sequencing were performed in samples of 980 patients. Appropriate statistical methods were adopted to assess HBV genetic features and its clinical association. It was found that the prevalent HBV genotype in northwestern Chinese patients was HBV/C (61.33%), followed by HBV/B (36.63%). In RT region, in addition to the reported nucleoside analogue- (NA-) resistance missense mutations, new silent mutations at rt169 and rt180 were found to raise the risk of HCC in patients with HBV/C. And the heterozygous mutation status of rt169/rt180 was associated with the increased risk of both HCC and NA resistance (OR > 1, *P* < 0.01) regardless of HBV genotypes. In BCP-PreC region, multiple mutations and combinations, especially at nt 1762/1764 and nt 1896/1899, were characterized to be the causes of spurious HBeAg negativity and liver function injury, as well as the risk factors for HCC progression (*P* < 0.01). Additionally, a novel mutation at nt1799G>C was likely found to increase the risk of HCC in patients with HBV/B. These findings revealed an association between HBV genotypes and HBV genetic mutations in RT and BCP-PreC region and progression of hepatitis B. It would be helpful for risk evaluation and diagnostic improvement based on these genetic features.

## 1. Introduction

Hepatitis B virus (HBV) poses a great endemic threat worldwide. In China, there are roughly 100 million HBV-infected patients and virus carriers [1, 2], one-third of whom have chronic hepatitis B, and each year approximately 500,000 people die prematurely from liver function failure, liver cirrhosis (LC), hepatocellular carcinoma (HCC), and other complications secondary to HBV infection [3].

HBV is an enveloped virus. It has a partially double-stranded circular DNA genome which carries four extensively overlapping open-reading frames, namely, C, X, P, and S. Gene C that contains the sequences of precore (preC) and core proteins encodes hepatitis B e antigen (HBeAg) and hepatitis B core antigen (HBcAg). X encodes transactivating protein X (HBx). P encodes DNA polymerase protein with reverse transcriptase (RT), and S encodes three surface antigen proteins (HBsAg). Because of drug pressure and DNA polymerase lacking proofreading capability, spontaneous mutations frequently occur, which may contribute to diverse clinical phenotypes, including the development of antiviral resistance and progression to HCC. For example, the RT region harbors multiple mutation sites associated with antiviral drug resistance [4]. Mutations within the basal core promoter (BCP; a regulatory sequence upstream of gene C) and the preC/C regions are correlated with HBeAg expression, virus loading, and progression of chronic hepatitis B to HCC [5, 6].

During the worldwide evolution of HBV virus, geographic and population differences and selective pressure from treatment have resulted in ten known HBV genotypes (A to J) containing sequence variations greater than 8% of the entire genome and reflecting distinctive geographical distributions [7–9]. Different HBV genotypes are associated with distinct clinical phenotypes and prognosis. For example, some research found that, compared to genotypes B and C, genotype A preferentially progresses to chronic hepatitis B [10], genotype D is closely associated with acute hepatitis [11], genotype B more readily develops antiviral resistance than genotype C, and genotype C takes a more aggressive disease risk of HCC than genotype B [12, 13].

HBV infection is a critical incentive for chronic hepatitis B (CHB), LC, and HCC. Different genotypes and genome mutations of HBV have been found to be related to these liver diseases and progression. However, our knowledge on HBV genotypes and genetic variations in HBV patients is quite limited and controversial because of conflicting data from different geography [14–16]. We lack the information that could enable us to recognize how HBV genotypes, naturally occurring mutations, induced mutations, and progression of HBV-infected diseases are related, how to estimate antiviral resistance based on HBV genomic sequences, or how to predict prognosis in chronic hepatitis B. To address these issues, we conducted our study on a cohort of HBV-infected northwest Chinese patients within different clinical courses by determining the genotypes and hot spot mutations in RT and BCP-PreC regions and analyzing its association with HCC development and antiviral resistance.

## 2. Materials and Methods

### 2.1. Patients, Samples, and Groups

A total of 980 patients were recruited in Xijing Hospital and Xi'an No. 323 Hospital in Xi'an, Shaanxi Province, from January 2012 to June 2016. 494 treatment-naive out-clinic patients diagnosed with HBV infection were included in the treatment-naive group, who were positive for HBsAg, with a serum HBV DNA load of ≥10^3^ copies/ml and without previous diagnosis of HBV infection or history of anti-HBV treatment (namely, not HBV carriers). 53 inpatients diagnosed with chronic hepatitis B were included in the nucleoside analogue- (NA-) resistant group. They had been treated with NA such as Lamivudine, Adefovir, or Entecavir for at least one year and had developed antiviral resistance. Its characteristic was that the serum HBV DNA load rebounded back to >10^3^ copies/ml, and alanine aminotransferase (ALT) and/or aspartate aminotransferase (AST) serum levels exceeded 40 U/L. 433 patients with HCC were included in HCC group, who were diagnosed by the clinician according to published guidelines [17] and underwent liver surgery at Xijing Hospital (Xi'an, Shaanxi Province, China) from January 2010 to November 2015. As control, the above 547 patients consisting of treatment-naive patients and NA-resistant patients were included in non-HCC group. Peripheral blood was collected from the patients in all groups, and paraffin-embedded liver tissues were obtained from the HCC patients. This study was approved by the ethics committee of 323 Hospital (Xi'an, Shaanxi Province, China) and Xijing Hospital (Air Force Military Medical University, China), and informed consent was obtained from each patient and was conducted according to the 1975 Declaration of Helsinki.

### 2.2. Primer Design

We obtained 53 complete genome sequences for HBV genotypes A to H from GenBank. These sequences were aligned and compared using Clustal X software (SFI, Ireland) to characterize the S, P, BCP-PreC, and C (BCP-PreC/C) regions of different HBV genotypes. Two pairs of nested Polymerase Chain Reaction (PCR) primers were then designed for each of the P and BCP-PreC/C genes of HBV genotypes A-H (Supplementary [Supplementary-material supplementary-material-1]). These were compared using Clustal X software to 3222 HBV whole genome sequences from Chinese patients in the database to ensure that the primers would work on highly conserved regions of the HBV genome in the Chinese population.

### 2.3. HBV DNA Extraction and Amplification

HBV DNA was extracted from the serum using a TIANamp Blood DNA Mini kit and from paraffin-embedded tissues using a TIANquick FFPE DNA kit (Tiangen Biotech., Beijing, China) in accordance with the manufacturer's instructions. Extracted DNA was then amplified using nested PCR on a Veriti Thermal Cycler (ABI, Oyster Bay, NY, USA). The first-round of PCR was performed using the outer primers, and the program was as follows: denaturation at 95°C for 5 min; 40 cycles at 94°C for 30 s, 52°C for 50 s, and 72°C for 50 s, followed by extension at 72°C for 5 min. The second round of PCR was performed using the inner primers under the same PCR conditions. The amplified fragments were sequenced by Genewiz (Suzhou, China).

### 2.4. HBV Genotyping and Sequence Analysis

HBV genotypes were determined by both phylogenetic trees comparing with standard sequences in GenBank database (Supplementary [Supplementary-material supplementary-material-1]) and HBV Liner software (an offline, HBV genotyping tool we developed previously, Chinese software copyright #1024141, patent #ZL 2011 10118281.7) on sequences amplified from HBV S, P, and BCP-PreC/C genes of each patient. Mutations were identified on those sequences using a Sequence Scanner (Applied Biosystems, CA, USA) and HBV Drug Guide software (an offline, HBV sequence data reading tool we developed previously, Chinese software copyright #1024137, patent #ZL 2011 10152837.4).

### 2.5. Statistical Analysis

STATA MP 14.0 was used to perform all statistical analysis. G^*∗*^power 3.1 was used to calculate the power (1 − *β*) of all tests between treatment-naive group and NA-resistant group. Continuous data fitting normal distribution were shown as mean ± sd and compared using Student's *t*-test or one-way ANOVA, and post hoc multiple comparisons were conducted using Tukey's method. Other continuous data were shown as median (quartile 25, quartile 75) and compared using Mann–Whitney *U* test. Categorical data were compared using chi-squared test or Fisher's exact test. Odds ratio (OR) and 95% confidence intervals (CI) were evaluated using logistic regression. Stepwise logistic regression was used to evaluate independent factors associated with HCC development. All statistical tests were two-sided and the difference was considered significant when the *P* value was less than 0.05 unless a particular statement.

## 3. Results

### 3.1. Patients Characteristics and HBV Epidemic Feature

The demographic and clinical characteristics of all patients recruited are shown in Table 1, including 433 HCC patients and 547 non-HCC patients which consisted of 494 treatment-naive patients and 53 NA-resistant patients. Overall, ages of included patients were 52.40 ± 12.10 years. There was no significant difference of age, gender composition, and alanine aminotransferase (ALT) level either between HCC patients and non-HCC patients or between treatment-naive patients and NA-resistant patients. HBV DNA was more abundant in non-HCC patients (*P* < 0.01). Infected HBV genotypes were identified by DNA sequencing and analyzed using NCBI Genotyping Tool and HBV Liner software. It was shown that HBV genotype C (HBV/C) was the most prevalent in northwest China and accounted for 61.33% of all patients recruited, followed by genotype B that accounted for 36.63%. Genotype D was rare and only accounted for 2.04%. Other genotypes such as A, E, F, G, and H were undetected. For the main genotypes, B2 and C2 subtypes were predominated as shown in Table 1. Of note, it showed a higher proportion of patients with HCC in those infected with HBV/C than in those with HBV/B (*P*=0.007), suggesting that patients with HBV/C were at a higher risk of developing into HCC. However, HBV genotypes were found not to be correlated with NA resistance (*P*=0.68).

### 3.2. Silent Mutations in HBV Polymerase Gene Increased the Risk of HCC

Polymerase gene reverse transcription conserved region (RT region) in HBV genome harbored mutations associated with HCC and NA resistance [18]. We amplified the RT region that encoded reverse transcriptase at rt155-rt330 and analyzed it using ABI Sequence Scanner and HBV Drug Guide software in the 980 patient samples. Mutations identified are listed in Supplementary [Supplementary-material supplementary-material-1]. In addition to the common hot spot missense mutations of rt204, rt236, rt202, rt250, and rt181 related to NA resistance, we noticed that rt169 and/or rt180 mutations predominantly happened in HBV/B and some in HBV/C-infected patients, and these mutations were mostly the silent mutations. The silent mutation at rt169 (41.94% of total 980 samples) was from ATT to ATA in most cases and sometimes to ATC, which did not change the encoded amino acid, isoleucine (Ile). The silent mutation at rt180 (43.88% of total 980 samples) was mainly from CTG to TTG and sometimes to CTC, which did not change the encoded amino acid, leucine (Leu). These two silent mutations usually coincided with each other, accounting for 41.63% in 980 samples. Logistic regression analysis was conducted to report crude OR and adjusted OR for the confounding effects of age, gender, and antiviral therapy. It revealed that mutations at rt169 and/or rt180 raised the risk of HCC in patients with HBV/C (Table 2, all OR > 1.0, *P* < 0.01).

Additionally, we noticed that the sequencing peaks at rt169 and rt180 in DNA sequencing map were overlapped with mutant and wild-type deoxynucleotides from PCR products, respectively (Supplementary [Supplementary-material supplementary-material-1]). We called that mutant type heterozygous. Accordingly, we collected these samples and cloned the PCR products into *E. coli* and randomly sampled the monoclonal for DNA sequencing. It was found that homozygous mutations predominated in non-HCC group (accounting for 84.34%), while heterozygous mutations at rt169 or/and rt180 predominated in HCC group (accounting for 81.77%). Logistic regression analysis showed that heterozygous mutations at rt169 or/and rt180 were associated with the risk of HCC pathogenesis (Table 3, OR = 24.15, *P* < 0.01). Similarly, heterozygous mutations at these sites also predominated in NA-resistant group (accounting for 61.54%) and were shown to increase the risk of NA resistance (Table 3, OR = 13.27, *P* < 0.01).

Besides, other mutation sites likely involved in drug resistance or HCC were observed with less frequencies. That is, mutations at rt204 (Met>Val/Ile/Thr) and rt202 likely increased the risk of NA resistance, and mutations at rt236 (Asn>Thr/His) and rt202 (Ser>Ile/Thr/Gly/Asn), rather than at rt204, seemed to increase the risk of HCC (*P* < 0.01 as shown in Supplementary [Supplementary-material supplementary-material-1]).

### 3.3. Multiple Mutations in HBV BCP-PreC Region

Given the importance of BCP, precore, and core gene in hepatitis progression and resistance to interferon treatment [4], we examined the mutations within BCP-PreC/C region. Seven most frequent mutations found in BCP-PreC region were shown in Supplementary [Supplementary-material supplementary-material-1]. All of them at nucleotide (nt) positions 1764, 1762, 1896, 1753, 1899, 1766, and 1768 were apparently abundant in patients with HBV/C and in HCC patients, while these mutations were insignificant for NA resistance.

We found that double mutations at nt 1762/1764 were most frequently observed in HCC patients compared with non-HCC patients consistently (Table 4, OR = 2.49, *P* < 0.01). Another double mutations at nt 1896/1899 were of intermediate frequency and also remarkably abundant in HCC patients (Table 4, OR = 5.68, *P* < 0.01). Stepwise logistic regression analysis revealed that double mutations at nt 1762/1764 and at nt 1896/1899 were independent factors that contributed to HCC development (Table 4, all *P* < 0.01). Interestingly, our data showed that HCC patients with mutations at nt 1762/1764 were highly susceptible to have coincident mutations at 1896 and/or 1899. The mutations coincident with the mutation at nt 1899 greatly contributed to HCC development as shown in Table 4 (*P* < 0.01), suggesting that the mutation at nt 1899 served an important role in some way. Triple mutations at nt 1762/1764/1753 and nt 1896/1899/1753 were also frequently occurred in HCC patients, but stepwise logistic regression analysis showed that they were not independent factors (Table 4).

Moreover, liver function injury with signs of alanine transaminase (ALT) and aspartate transaminase (AST) over 40 U/L were more frequently observed in patients with HBV/C (74.13%) and less frequently in patients with HBV/B (25.87%) (OR = 3.05, *P* < 0.01, Supplementary [Supplementary-material supplementary-material-1]). Mutations at nt 1762, nt 1764, and both in the BCP region, but not at any other sites, were found to be significantly correlated with liver injury (OR > 1.0, *P* < 0.05, Supplementary [Supplementary-material supplementary-material-1]).

### 3.4. Multiple Mutations in BCP-PreC Region Were Involved in HBeAg Seroconversion

HBeAg expression is an indicator for active viral replication. Several studies have reported that mutations within BCP-PreC/C are often associated with abnormal termination of HBeAg expression, probably because amino acid coding changes resulting from these mutations would result in HBeAg transcription dysfunction and the mistakes in HBeAg translation and posttranslational processing. However, this kind of HBeAg negativity does not represent the improvement of the disease treatment outcome but is a sign of the progress [19–21]. To examine if mutations we found would lead to spurious HBeAg negativity in this northwestern Chinese population, we stratified 336 patients in the treatment-naive group, whose HBeAg data were available, into three groups: control group that harbored samples without any mutations of nt 1762/1764/1896/1899, BCP core mutant group that included samples with nt 1762/1764 double mutation only, and a coincident mutant group that included samples with nt 1762 and/or nt 1764 mutations accompanied by nt 1896 and/or nt 1899 mutations. Patients with HBeAg value less than 1.0 S/CO was considered as HBeAg-negative. It was shown that the HBeAg-positive proportion was higher in the control group (67.74%), while the HBeAg-negative proportion predominated in BCP core mutant group (53.13%, *P* < 0.05) and coincidence group (80.09%, *P* < 0.01) as shown in Table 5. It was indicated that patients with HBeAg-negative features and mutations at nt 1762/1764/1896/1899 in BCP-PreC region were likely to promote disease progression rather than to be in remission stage.

### 3.5. Mutation of 1799G>C in HBV/B Was Involved in HCC Progression

Although many studies found that patients with HBV/C had an increasing risk of HCC, the risk mutation patterns in patients with HBV/B remained uncertain. By DNA sequencing and analysis, we found that a point mutation of 1799G/C frequently existed in HBV BCP region. To identify which kind of nucleotides represented a major/wild type, we collected data in the NCBI database regarding HBV strains infecting Chinese populations and found that the nucleotide at 1799 varies among HBV genotypes. For HBV/B, the major nucleotide at 1799 site was guanine (G, 86.85%) and could be regarded as the wild type; the minor nucleotide was cytosine (C, 13.15%) and represented a mutant type. For HBV/C, C was the major nucleotide (97.41%), which was consistent with our findings that 1799C was prevalent in all patients with HBV/C. In patients infected with HBV/B, most of the non-HCC patients have wild type 1799G (65.91%), but a great proportion of HCC patients with 1799G>C mutation (89.93%) are observed as shown in Table 6, and regression analysis revealed that 1799G>C in patients with HBV/B raised the risk of HCC (OR = 17.26, *P* < 0.01, Table 6). Additionally, we examined the association between 1799G>C mutation and serum HBeAg index in 162 treatment-naive patients with HBV/B, whose HBeAg data were available. It showed that patients with the 1799G>C mutation were more frequent to become HBeAg-negative (OR = 5.47, *P* < 0.01). These findings indicated that 1799G>C mutation might be a promising clinical indicator of HCC progression in patients with HBV/B.

## 4. Discussion

In this study, we characterized HBV genotypes and hot spot mutations in RT and BCP-PreC/C regions among HBV-infected patients in northwest China and analyzed their associations with distinct disease features including the development into HCC, development of resistance to antiviral treatment, liver function injury, and serum HBeAg negativity.

Mutations in polymerase gene RT region in HBV genome have been known to be associated with antiviral drug resistance, since nucleotide analogues such as lamivudine, entecavir, and telbivudine function as reverse transcriptase inhibitors by mimicking natural nucleotides and integrating within the DNA molecules to interfere with viral replication [18]. It has been reported that rt169 Ile>Thr is the primary mutation responsible for resistance to Entecavir, and rt180 Leu>Met is a compensatory mutation for resistance to entecavir, lamivudine, and telbivudine [1, 22]. In our study, we found a kind of silent mutations at rt169 and rt180, instead of those missense mutations, predominated in patients and seemed not to contribute to the risk of HCC or NA resistance. However, by further analysis in patients with each HBV genotype, it revealed that the rt169/rt180 silent mutations raised the risk of HCC in patients with HBV/C. As we know, RT and S gene share the same part of DNA sequences. By comparison, we found that the silent mutations of rt169 and rt180 in RT caused missense mutations in S gene. That is, mutation at rt169 in RT resulted in mutation of 161 Phe>Thy/Ser (F161Y/S) in HBsAg, and mutation at rt180 in RT resulted in mutation of 172 Trp>Ser (W172S) in HBsAg. HBV S gene encodes three envelop proteins: preS1, preS2, and S proteins. PreS/S variants are often identified in hepatitis B infected CHB, LC, and HCC and may contribute to the development of progressive liver damage and hepatocarcinogenesis [23, 24]. Pre-S deletion, pre-S mutation, S mutation, and splice variants may use different routes to cause liver diseases leading to accumulation of envelop protein and viral particles and induction of ER stress and consequently promote HBV immune escape and HCC development [14, 25]. There are studies that have reported that F161Y and W172S mutations in S were closely related to immune escape and promoted hepatitis B progression [26, 27]. This may explain why rt169/rt180 silent mutation in our study related to a high risk of HCC progression and NA resistance. Moreover, by monoclonal sequencing and logistic regression analyzing, we observed another important phenomenon that mutations at rt169 and rt180 as heterozygosity were significantly associated with increased risk of NA resistance and HCC progression. This existed not only in patients with HBV/C but also in those with HBV/B, reminding us that drug pressure and disease progression probably induced genetic drift and resulting in a high quasi-species complexity and diversity of HBV strains.

In consistent with previous findings that mutation at rt204 accounts for antiviral resistance to lamivudine, entecavir, or telbivudine [28], we found that rt204 mutation was particularly abundant in NA-resistant patients with HBV/C. The mutation detected for rt204 led to three possibilities, valine (GTG), isoleucine (ATT), or serine (AGT/C), which were also consistent with antiviral-resistant mutations reported for this site [18, 29]. Another site for antiviral resistance we found was rt202, which also represented resistance to entecavir in Asian population [30]. Mutation at rt236 was responsible for resistance to adefovir dipivoxil in Caucasian [31], but here it seemed to contribute to HCC instead of antiviral resistance in Chinese population. However, the result is preliminary due to the limited case samples in nucleoside analogue- (NA-) resistant group, but it still provides some clues because the statistical power of all tests listed in tables was examined and found over 0.8.

The BCP-PreC/C region is another important genetic area not only because it encodes proteins that are integral for HBV structure and functions but also because it is vulnerable to mutations that are associated with liver function injury and progression to HCC [5, 32]. Mutations at nt 1762, 1764, 1766, and 1768 located in BCP region have been reported to be associated with interferon-resistance and liver function injury [33]. Consistently, our data showed that double mutations at nt 1762/1764 were a significant risk factor of liver injury featured by elevated serum ALT and AST levels. Many studies and meta-analysis showed double mutation at nt 1762/1764 increased the risk of HCC, as well as mutation at nt 1896 in PreC region [6, 34]. PreC/C responses to encoding HBcAg and HBeAg. Mutation at nt 1896 had been reported to promote the progression to HCC in western Asian population [35]. Though some studies reported that mutation at nt 1899 was nonsignificant to HCC development, our data revealed that relative high frequent mutations of nt 1896, nt 1899, and nt 1896/1899 double mutations in PreC apparently increased the risk of HCC in northwest Chinese population, which might be attributed to the correlation between mutation at nt 1899 and liver failure [36]. Furthermore, we found a coincidence between double mutation at nt 1762/1764 and nt 1896/1899, and the quadruple mutation was shown as a risk factor of HCC (OR = 5.68). Besides, we revealed the strong association between multiple mutations at nt 1762/1764/1896/1899 and HBeAg seroconversion, which was supported by the findings that 1762/1764 double mutations repress HBeAg precursor expression [37]. The 1896G>A nonsense mutation terminates HBeAg expression by creating a stop codon [33], and the 1899G>A results in a missense mutation that inhibits the recognition and cleavage of HBeAg precursor by related enzymes [38], which eventually led to intracellular accumulation of HBeAg and its negativity in serum [39, 40]. Therefore, it could be concluded that those multiple mutations in RT and BCP-PreC region were not able to detect HBeAg and never impeded HBV replication, and therefore this led to a spurious HBeAg negativity. These findings may be helpful to guide the accurate assessment of progression of chronic hepatitis B when serum is HBeAg-negative and to prevent the misconception that HBeAg negativity represents reduced viral replication and disease progression has been controlled.

Another finding was the correlation between HBV/B infection and mutation at nt 1799G>C that was located in BCP region and overlapped with the open reading frame of HBV X gene. One study in Asian population reported that G1799C served an important role in HBeAg turning negative [41]. Our data revealed that mutation at nt 1799 was closely related to HCC (OR = 17.26) and HBeAg negativity (OR = 5.47) in patients with HBV/B. We speculate that this mutation may affect HBx gene expression, structure, and function. The underlying mechanisms, however, should be further studied.

In conclusion, by profiling the HBV genotypes and hot spot mutations among northwest Chinese patients, we identified a series of mutations in RT and BCP-PreC region and revealed that quasi-species complexity and diversity of HBV greatly contributed to HBV-related disease progression. Our findings include the confirmation of the prevalent of HBV/B and HBV/C in China, NA resistance related common mutations in RT region, and disease progression associated mutations in BCP-PreC region. We also revealed some new mutations and combinations with disease progression such as rt169/rt180 silent mutation and heterozygous mutation, nt 1896/1899 double mutation and their combination with nt 1762/1764 double mutation, and HBV/B genotype specific nt 1799 mutation. These findings together with previous reported variants proposed promise for early prediction of specific hepatitis B subtype progressing toward HCC and for selection of treatment strategy in the case of HBeAg seroconversion with high HBV DNA copies.

## Figures and Tables

**Table 1:** Demographic and serological features of each group.

	HCC	Non-HCC	*P* ^1^	Non-HCC		*P* ^1^
				Treatment-naive	NA-resistant	
*n*	433	547		494	53	
Age^2^	53.56 ± 11.27	52.24 ± 11.86	0.08	51.36 ± 12.03	50.21 ± 10.27	0.50
Gender ratio (male/female)	1.62	1.43	0.37	1.47	1.12	0.35
HBV DNA (IU/ml, log10)^3^	2.72 (1.07, 4.92)	3.68 (3.04, 6.17)	<0.01	3.92 (2.41, 5.25)	3.61 (1.58, 5.46)	0.79
ALT (IU/L)^3^	155.60 (43.10, 391.50)	79.50 (28.90, 326.70)	0.41	76.60 (31.50, 283.50)	62.00 (42.80, 254.50)	0.59
Genotype			<0.01			0.68
B	139 (32.10%)	220 (40.22%)	0.007	198 (40.08%)	22 (41.51%)	
C	286 (66.05%)	315 (57.59%)		286 (57.89%)	29 (54.72%)	
D	8 (1.85%)	12 (2.19%)		10 (2.02%)	2 (3.77%)	
Subtype						
HBV/B						
B1	7 (5.04%)	14 (6.36%)	0.602	2 (1.01%)	0 (0.00%)	1.000
B2	132 (94.96%)	206 (93.64%)		196 (98.99%)	22 (100.00%)	
HBV/C						
C1	42 (14.69%)	52 (16.51%)	0.539	33 (11.54%)	6 (20.69%)	0.149
C2	244 (85.31%)	263 (83.49%)		253 (88.46%)	23 (79.31%)	

^1^Student's *t*-test or Mann–Whitney *U* test was used for continuous data, and chi-square or Fisher's exact test was used for categorical data. ^2^Data are shown as mean ± standard deviation. ^3^Data are shown as median (quartile 25, quartile 75).

**Table 2:** HBV genotypes differed the risks of rt169/rt180 mutations in HCC versus non-HCC.

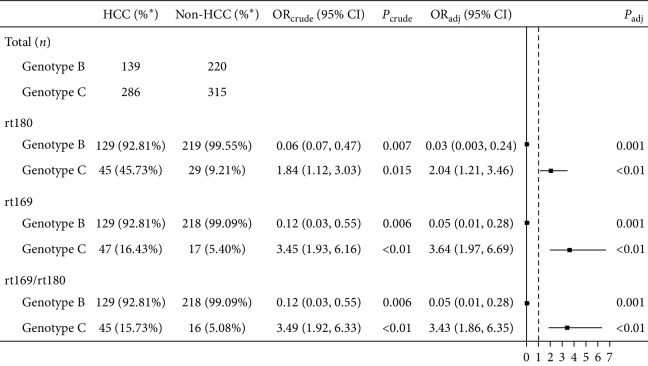

^*∗*^Percent of rt169/rt180 mutations.

**Table 3:** Mutant types of rt169/rt180 in each group.

	Total	rt169/rt180 (%)		OR (95% CI)	*P*
		Heterozygous	Homozygous		
HCC versus non-HCC					
HCC	181	148 (81.77%)	33 (18.23%)	24.15 (14.58, 40.03)	<0.01
Non-HCC	249	39 (15.66%)	210 (84.34%)		
NA-resistant versus treatment-naive					
NA-resistant	26	16 (61.54%)	10 (38.46%)	13.27 (5.35, 33.59)	<0.01
Treatment-naive	223	24 (10.76%)	199 (89.24%)		

**Table 4:** Multiple mutation patterns in HBV BCP-PreC region.

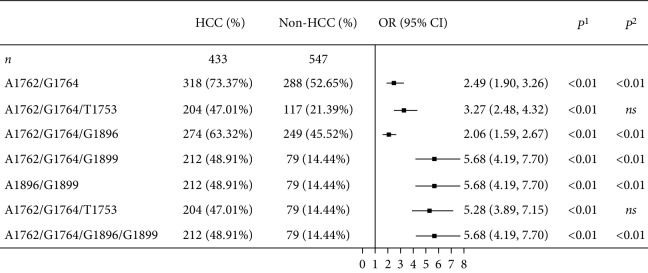

^1^ *P*: likelihood ratio test for logistic regression. ^2^*P*: Wald test for stepwise logistic regression. *ns*: nonsignificant.

**Table 5:** Patients with nt 1762/1764/1896/1899 mutations vulnerable to HBeAg negativity.

	HBeAg		OR (95% CI)	*P*
	Negative (%)	Positive (%)		
Control	30 (32.26%)	63 (67.74%)		
BCP mutant core (A1762/G1764 double only)	17 (53.13%)	15 (46.88%)	2.38 (1.05, 5.40)	0.038
Coincidence (A1762 and/or G1764/G1896 and/or G1899, triple/quadruples)	169 (80.09%)	42 (19.91%)	8.45 (4.87, 14.66)	<0.01

**Table 6:** 1799G>C mutation in patients with HBV/B.

G1799C		WT	OR (95% CI)	*P*
HCC susceptibility (*n* = 359)				
HCC	125 (89.93%)	14 (10.07%)	17.26 (9.30, 32.05)	0.001
Non-HCC	75 (34.09%)	145 (65.91%)		
HBeAg (treatment-naive, available *n* = 162)				
Negative	28 (27.72%)	73 (72.28%)	5.47 (1.81, 16.48)	0.001
Positive	4 (6.56%)	57 (93.44%)		

## Data Availability

All data used to support the findings of this study are included within the article and the supplementary information files. The raw data in this study are restricted by the Independent Ethics Committee, Xijing Hospital, China, in order to protect patient privacy. Data are available from Dr. Wei Wang (e-mail: wangwei_fmmu@163.com) for researchers who meet the criteria for access to confidential data.

## Abbreviations

HBV: Hepatitis B virus
LC: Liver cirrhosis
HCC: Hepatocellular carcinoma
preC: HBV precore protein
HBx: HBV transactivating protein X
HBsAg: HBV surface antigens
HBeAg: HBV e antigens
BCP: Basal core promoter
ALT: Alanine aminotransferase
AST: Aspartate aminotransferase
OR: Odds ratio
CI: Confidence interval
RT: Reverse transcriptase
NA: Nucleoside analogue.

## Supplementary Materials

Supplementary Materials Table S1: GenBank Accession IDs*∗* for the whole genome sequences of human hepatitis B virus (HBV) genotypes A to H used in this study. Table S2: primer sequences for nested polymerase chain reaction (PCR) of the P and BCP-PreC/C genes of the hepatitis B virus (HBV) genome. Table S3: mutations within RT region. Table S4: mutations within BCP-pre C region. Table S5: liver function injury of patients with mutations in BCP-pre C region. Figure S1: DNA sequencing map of mutations at rt169 and rt180 in P region of hepatitis B virus (HBV).
