# Supplementary material for: Genotypes and Hot Spot Mutations of Hepatitis B Virus in Northwest Chinese Population and Its Correlation with Diseases Progression
Source: Biomed Res Int. 2019 Dec 10;2019:3890962. doi: 10.1155/2019/3890962 (PMC6925797; doi:10.1155/2019/3890962)
Supplement: Supplementary Materials — Table S1: GenBank Accession IDs∗ for the whole genome sequences of human hepatitis B virus (HBV) genotypes A to H used in this study. Table S2: primer sequences for nested polymerase chain reaction (PCR) of the P and BCP-PreC/C genes of the hepatitis B virus (HBV) genome. Table S3: mutations within RT region. Table S4: mutations within BCP-pre C region. Table S5: liver function injury of patients with mutations in BCP-pre C region. Figure S1: DNA sequencing map of mutations at rt169 and rt180 in P region of hepatitis B virus (HBV). [file 3890962.f1.doc]

**Table S1. GenBank Accession IDs^*^ for the whole genome sequences of human Hepatitis B virus (HBV) genotypes A to H used in this study**

| **HBV Genotype** | **GenBank ID** |
| --- | --- |
| A | X51970、X70185、AB064314、X02763、AF090842 |
| B | D00329、AF100309、AB033554、D00330、AB073858、AF121244、AF121249、D23678、D23679、AB010289、AB073846、AB602818 |
| C | X04615、AB014381、AB033556、AB014378、AB014393、AF068756、AF223960、AF458664、D23683、AF241411、AY040627、AY123041 |
| D | X65259、M32138、X85254、X02496、AF121239、AF121242、AJ344116、X97848、Y07587 |
| E | X75657、AB032431、X75664 |
| F | X69798、AB036910、AF223962、AF223964、AF223965 |
| G | AF160501, AB064310, AF405706, AB056513 |
| H | AY090454, AY090457, AY090460 |

* Underlined IDs refer to those used as reference sequences for the corresponding HBV genotypes

**Table S2. Primer sequences for nested Polymerase Chain Reaction (PCR) of the P and BCP-preC/C genes of the Hepatitis B virus (HBV) genome**

| **Primer** |  | **Sequence** | **Location** |
| --- | --- | --- | --- |
| P gene outer | Forward | 5’-ATGTGTCTGCGGCGTTTTAT-3’ | 380-399 |
|  | Reverse | 5’-ACTTTCCAATCAATAGG-3’ | 961-989 |
| P gene inner | Forward | 5’-GTTGCCCGTTTGTC-3’ | 466-479 |
|  | Reverse | 5’-ACTTTCCAATCAATAGG-3’ | 961-989 |
| BCP-pre C/C gene outer | Forward | 5’-TCGCTTCACCTCTGCAC-3’ | 1589-1605 |
|  | Reverse | 5’-AAAAAAGTCAGAAGGCAAAAA-3’ | 1955-1975 |
| BCP-pre C/C gene inner | Forward | 5’-AATGTCAACGACCGACCTT-3’ | 1682-1770 |
|  | Reverse | 5’-AAAAAAGTCAGAAGGCAAAAA-3’ | 1955-1975 |

BCP: basal core promoter; pre C: HBV pre core protein.

**Table S3. Mutations within RT region**

**
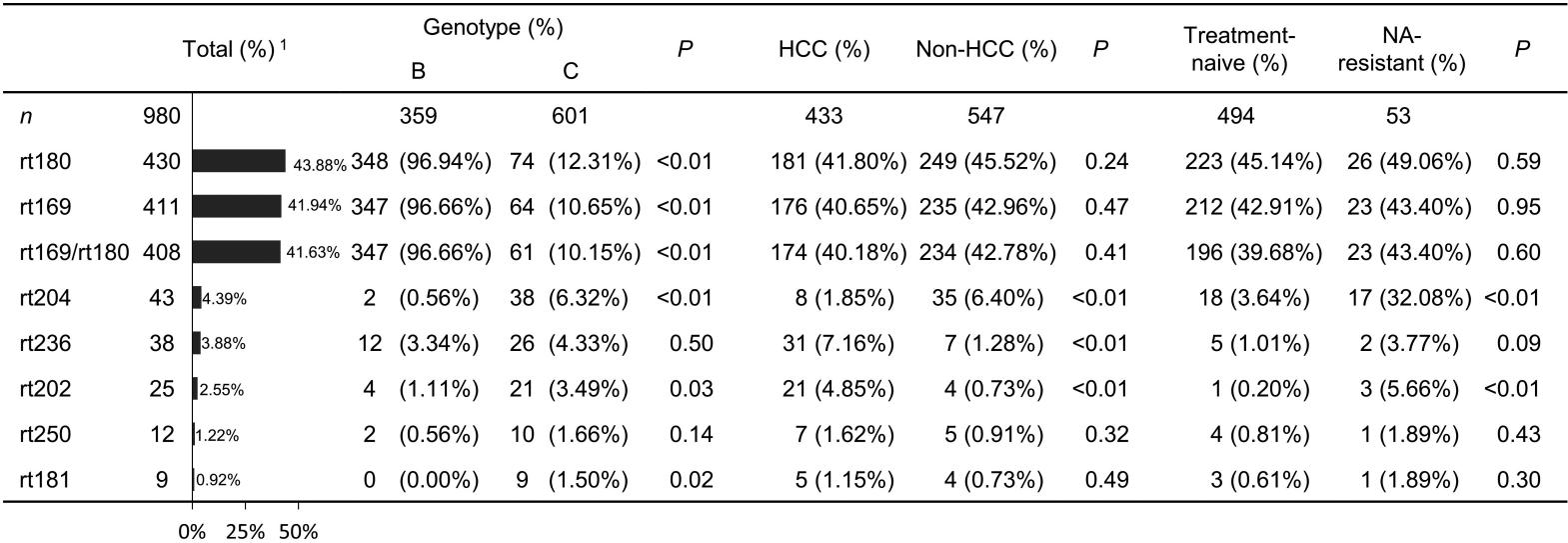
**

^1^Total number of samples in each type of mutations were presented as the sample number and its percentage with a bar chart.

**Table S4. Mutations within BCP-pre C region.**

**
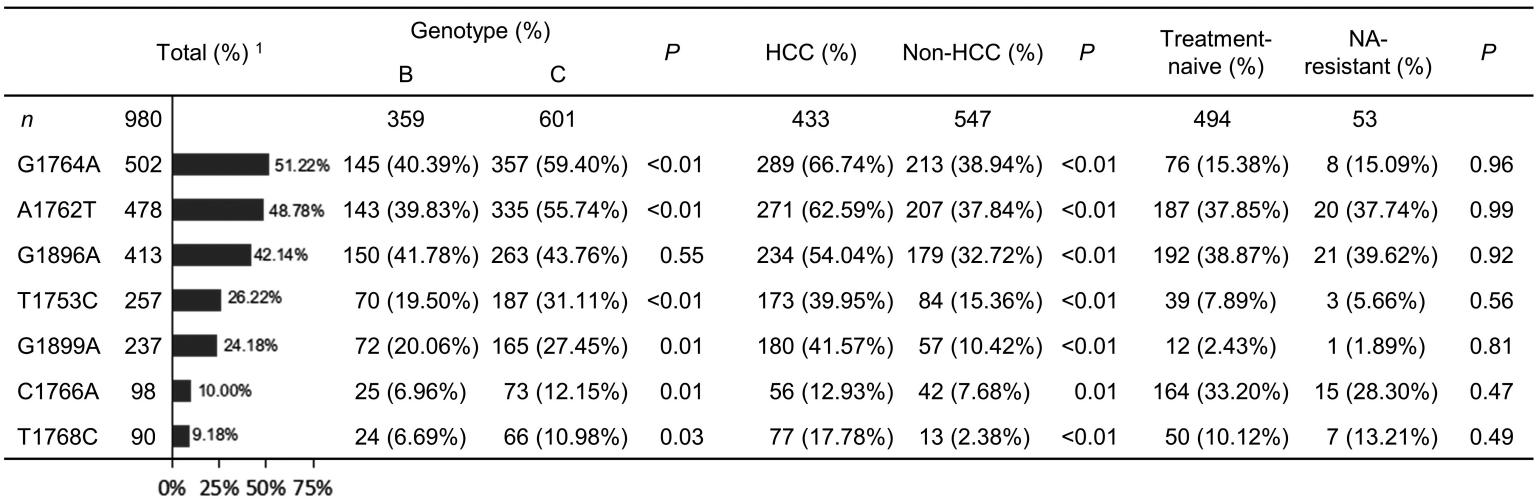
**

^1^Total number of samples in each type of mutations were presented as the sample number and its percentage with a bar chart.

**Table S5. Liver function injury of patients with mutations in BCP-pre C region.**


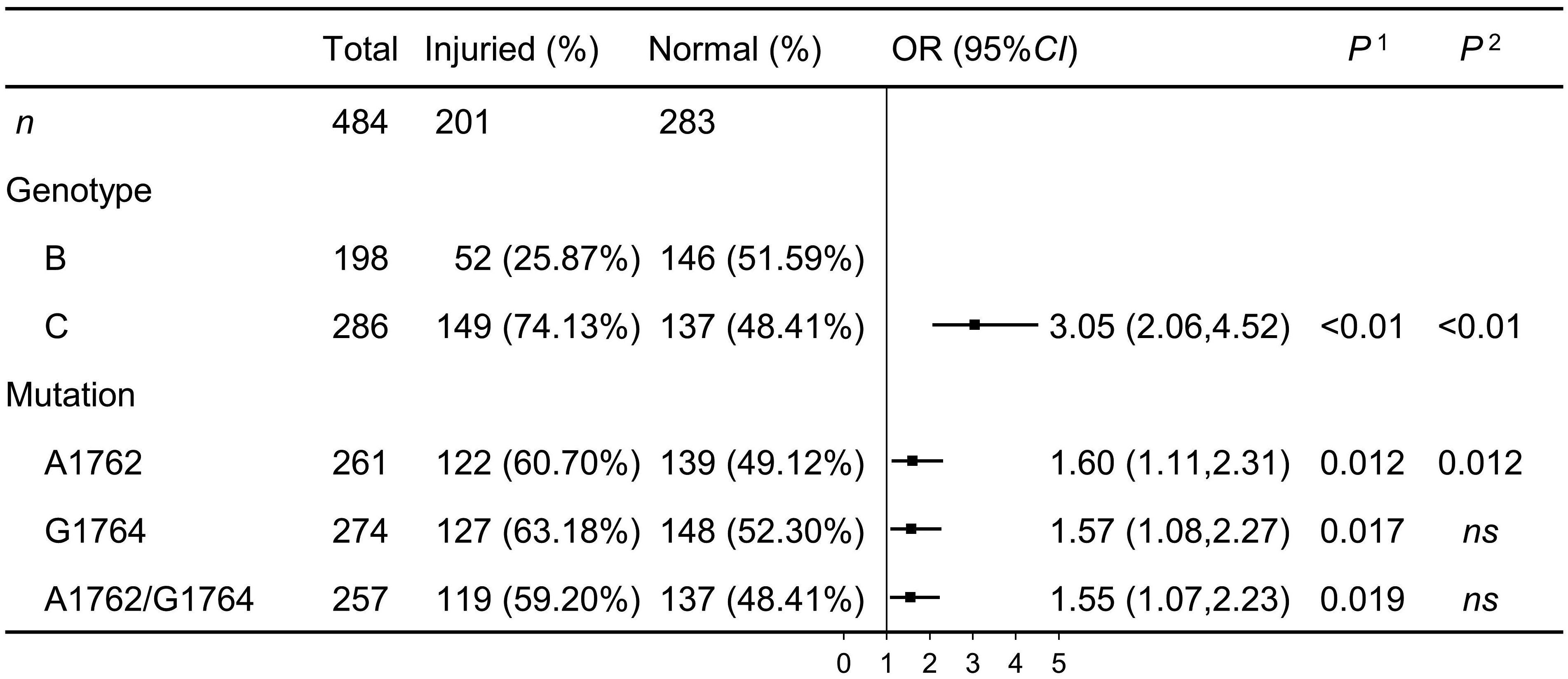


^1^*P* : likelihood ratio test for logistic regression.

^2^*P :* Wald test for stepwise logistic regression. The abbreviation *ns* stands for nonsignificant.


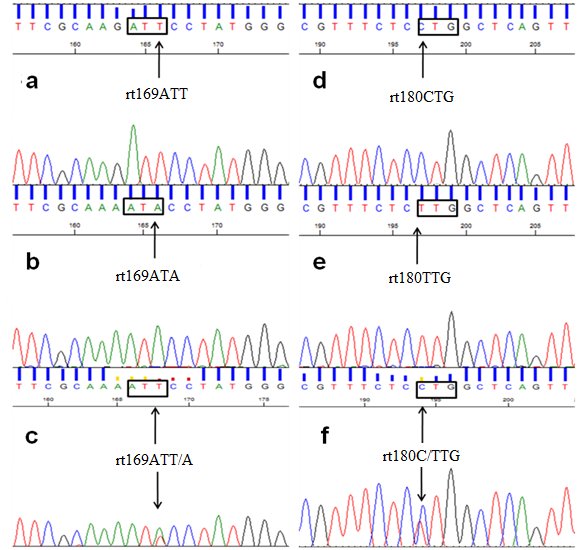


**Figure S1. DNA sequencing map of mutations at rt169 and rt180 in P region of Hepatitis B virus (HBV).**

The wild type sequences at rt169 (a) and rt180 (d) are ATT and CTG, respectively. In treatment-naive patients, most detected HBV strains contained homozygous mutations at these two sites, that is ATA (b) at rt169, and TTG (e) at rt180. In NA-resistant and LC/HCC patients, however, heterozygous mutations at both sites were observed, leading to overlap of both mutant and wild type sequencing peaks detected at both sites (heterozygous mutations, c and f).

NA, nucleoside analogue; LC, liver cirrhosis; HCC, hepatocellular carcinoma; rt, reverse transcriptase.
